# Persistent Pesticides in Human Breast Milk and Cryptorchidism

**DOI:** 10.1289/ehp.8741

**Published:** 2006-02-27

**Authors:** Ida N. Damgaard, Niels E. Skakkebæk, Jorma Toppari, Helena E. Virtanen, Heqing Shen, Karl-Werner Schramm, Jørgen H. Petersen, Tina K. Jensen, Katharina M. Main, the Nordic Cryptorchidism Study Group

**Affiliations:** 1 University Department of Growth and Reproduction, Copenhagen, Denmark; 2 Departments of Physiology and Pediatrics, University of Turku, Turku, Finland; 3 GSF-National Research Center for Environmental and Health, Institute for Ecological Chemistry, Neuherberg, Germany; 4 Department of Biostatistics, University of Copenhagen, Copenhagen, Denmark

**Keywords:** cryptorchidism, human breast milk, infants, persistent organochlorine pesticides

## Abstract

**Introduction:**

Prenatal exposure to some pesticides can adversely affect male reproductive health in animals. We investigated a possible human association between maternal exposure to 27 organochlorine compounds used as pesticides and cryptorchidism among male children.

**Design:**

Within a prospective birth cohort, we performed a case–control study; 62 milk samples from mothers of cryptorchid boys and 68 from mothers of healthy boys were selected. Milk was collected as individual pools between 1 and 3 months postpartum and analyzed for 27 organochlorine pesticides.

**Results:**

Eight organochlorine pesticides were measurable in all samples (medians; nanograms per gram lipid) for cases/controls: 1,1-dichloro-2,2-bis(4-chlorophenyl)ethylene (*p,p*′-DDE): 97.3/83.8; β-hexachlorocyclohexane (β-HCH): 13.6/12.3; hexachlorobenzene (HCB): 10.6/8.8; α -endosulfan: 7.0/6.7; oxychlordane: 4.5/4.1; 1,1,1-trichloro-2,2-bis(4-chlorophenyl)ethane (*p,p*′-DDT): 4.6/4.0; dieldrin: 4.1/3.1; *cis*-heptachloroepoxide (*cis*-HE): 2.5/2.2. Five compounds [octachlorostyrene (OCS); pentachlorobenzene, 1,1-dichloro-2,2-bis(4-chlorophenyl)ethane (*p,p*′-DDD); *o,p*′-DDT; mirex] were measurable in most samples (detection rates 90.8–99.2%) but in lower concentrations. For methoxychlor, *cis*-chlordane, pentachloroanisole (PCA), γ -HCH, 1,1-dichloro-2-(2-chlorophenyl)-2,2(4-chlorophenyl)ethane, *trans*-chlordane, α -HCH, and *o,p*′-DDE, both concentrations and detection rates were low (26.5–71.5%). Heptachlor, HCH (δ, ɛ ), aldrin, β-endosulfan and *trans*-heptachloroepoxide were detected at negligible concentrations and low detection rates and were not analyzed further. Seventeen of 21 organochlorine pesticides [*p,p*′-DDT, *p,p*′-DDE, *p,p*′-DDD, *o,p*′-DDT, HCH (α , β, γ ), HCB, PCA, α -endosulfan, *cis*-HE, chlordane (*cis*-, *trans*-) oxychlordane, methoxychlor, OCS, and dieldrin] were measured in higher median concentrations in case milk than in control milk. Apart from *trans*-chlordane (*p* = 0.012), there were no significant differences between cryptorchid and healthy boys for individual chemicals. However, combined statistical analysis of the eight most abundant persistent pesticides showed that pesticide levels in breast milk were significantly higher in boys with cryptorchidism (*p* = 0.032).

**Conclusion:**

The association between congenital cryptorchidism and some persistent pesticides in breast milk as a proxy for maternal exposure suggests that testicular descent in the fetus may be adversely affected.

Studies published during the last decades have indicated that the birth prevalence of cryptorchidism may have increased in some regions (Berkowitz et al. 1995; Boisen et al. 2004; John Radcliffe Hospital Cryptorchidism Study Group 1992; Pierik et al. 2004; Thong et al. 1998). Genetic factors may contribute to these findings. However, the short time interval of this increase suggests that environmental factors may be important (Sharpe and Skakkebæk 2003).

Several organochlorine pesticides can cause adverse effects in the male reproductive system in animals (Edwards et al. 2006; Vos et al. 2000). An increased incidence of cryptorchidism in male panthers has been attributed to endocrine-disrupting chemicals in the environment, such as 1,1-dichloro-2,2-bis(4-chlorophenyl)ethane (*p,p*′-DDE) (Facemire et al. 1995). *In utero* exposure of male rats and rabbits to dichlorodiphenyldichloroethylene (DDT) resulted dose dependently in reduced anogenital distance, hypospadias, cryptorchidism, and epididymal agenesis (Gray et al. 2001, 2004; Palmer et al. 2000; Wolf et al. 1999; You et al. 1998).

Increased rates of orchidopexy in areas with extensive pesticide use in agriculture have been reported (Garcia-Rodriguez et al. 1996). One study also found higher pesticide levels in fat tissue samples from boys operated on for cryptorchidism than in children who were operated on for other reasons (Hosie et al. 2000).

The aim of this study was to investigate, in a case–control study nested within a prospective cohort, whether individual *in utero* exposure to organochlorine pesticides estimated by measurements of maternal breast milk concentrations was associated with cryptorchidism among male children.

## Materials and Methods

We conducted a joint prospective, longitudinal birth cohort study in Finland (Turku University Hospital) and Denmark (National University Hospital at Copenhagen) from 1997 to 2001. In this study we aimed to describe regional prevalence rates and risk factors (lifestyle and exposure) for cryptorchidism by means of questionnaires and biologic samples (blood samples of mother and child, placentas, and one breast milk sample per child). The study was prospectively planned by both research groups as a joint venture in 1996. Recruitment and examinations were completely standardized. The cohort (antenatal recruitment and inclusion, inclusion criteria, and clinical examinations) has been described previously in detail (Boisen et al. 2004). All boys in the cohort were examined by a group of specially trained doctors for signs of cryptorchidism. The examination technique and definition of cryptorchidism developed by Scorer were applied (Scorer 1964). Standardization of the clinical procedures was achieved by repetitive workshops, and borderline cases were examined by two researchers from the national study groups. All boys were examined shortly after birth and again at 3 months of age. Boys born prematurely were examined at the expected date of delivery.

From the total biobank of breast milk samples, we included 65 samples from each country for organochlorine pesticide measurements. The number of samples was determined by funding. The samples represent 29 Danish and 33 Finnish cases defined as boys with cryptorchidism (unilateral or bilateral) at birth. Four Danish and 25 Finnish boys were still cryptorchid at 3 months of age, whereas the others (25/8) had spontaneous descent. Danish/Finnish controls (36/32), defined as boys without cryptorchidism at birth or 3 months, were included. In Denmark, the controls were selected randomly from the entire birth cohort of healthy boys. In Finland, the boys were selected prospectively by a case–control design in which the boys with cryptorchidism were matched to controls at birth for maternal parity, smoking (yes/no), diabetes (yes/no), gestational age (± 7 days), and date of birth (± 14 days). This design was chosen in Finland because of lack of sufficient funding to collect and store biologic samples from all. To ensure that all prospectively planned chemical analyses could be performed, only breast milk samples with a volume > 125 mL were included.

The study was conducted according to the Helsinki II Declaration (World Medical Association 2004) and was approved by the local ethical committees in both countries (Finland: 7/1996, Denmark: KF01-030/97) and the Danish Data Protection Agency (registration no. 1997-1200-074). The families were included after oral and written informed consent had been obtained from the parents.

Human breast milk samples were collected from 1 to 3 months postpartum in Denmark and from 1 to 2 months postpartum in Finland as successive aliquots. All mothers were given oral and written instructions to feed the baby first and then sample milk aliquots (hind milk) by manual expression into a glass or porcelain container, avoiding the use of mechanical breast pumps. The aliquots were frozen consecutively in a glass bottle (250-mL Pyrex glass bottle with a Teflon cap (1515/06D, Bibby Sterilin, Staffordshire, England) and stored in household freezers. The samples were delivered frozen to the hospital at the 3 months’ examination and stored at –20°C.

Exposure measurements in biologic samples from boys with congenital cryptorchidism at birth and controls were prospectively planned to include persistent and nonpersistent chemicals. Twenty-seven organochlorine compounds were selected by the following criteria: previous worldwide use, suspicion of endocrine-disrupting activity from animal and/or *in vitro* studies, and highly sensitive analytic methods available. As part of other substudies, the same breast milk samples were planned to be analyzed for other compounds with suspected endocrine-disrupting activity.

The selected breast milk samples were thawed at room temperature for 12 hr, heated, and shaken for 30 min at 37°C to homogenize the samples, and then divided into smaller aliquots and refrozen at –20°C until further chemical analysis. Extraction, cleanup, and analysis of organochlorine pesticides in the milk samples were based on a method that has been described in detail elsewhere (Shen et al. 2005). Milk samples (10 mL) were extracted with 250 mL of a mixture consisting of acetone and *n*-hexane (2:1 v/v) (Beek 2000). The milk extracts were collected in flasks weighed in advance and evaporated using a rotary vacuum evaporator (water bath at 45°C). After evaporation, the flasks were placed into an exicator until stable weight was achieved. The lipid content was calculated on wet weight basis. The residual was dissolved in toluene, and gel permeation-chromatography followed by sandwich cartridge cleanup was used to remove lipids and other interferences from the extract. Finally, the organochlorine pesticides were measured by high-resolution gas chromatography/high-resolution mass spectrometry quantified by an isotope dilution method.

## Statistical Analysis

Descriptive data of the mothers and the boys (anthropometric measurements) are reported as mean ± SD or number (percentage) (Table 1). We tested differences between boys with and without cryptorchidism by unpaired *t*-tests or chi-square tests. Descriptive statistics of pesticide concentrations are given as medians and ranges (minimum and maximum) because of skewed distributions. The sum of DDT metabolites was calculated as the sum of all six metabolites. We calculated the DDE(dichloro-diphenyldichloroethylene)/DDT ratio and the enantiomeric median ratio (ER) by simple division: *p,p*′-DDE/1,1,1-trichloro-2,2-bis(4-chlorophenyl)ethane (*p,p*′-DDT) and (+)-isomer concentration/(–)-isomer concentration for the individual compounds, respectively. We tested the differences between cryptorchid and healthy boys using the Mann-Whitney *U*-test. We tested the differences in pesticide levels between cryptorchid and healthy boys using a logistic regression model in which the pesticide level (log-transformed) and, in some analyses, country and other potential confounders were entered as covariates. This approach had the advantage of allowing the inclusion of cases and controls based on their pesticide levels in the analysis. This selection does not introduce bias in the estimation. *p*-Values were not corrected for multiple testing.

To determine whether a given measurement of a sample indicated the presence of a pesticide, both the limit of detection (LOD) and the limit of quantification (LOQ) were determined for every sample. We defined the LOD as three times the background noise of the analytic instrument. Samples with values below the LOD were nondetectable. Samples above LOD, in which the pesticide concentration could not be reliably quantified, were assigned a value below the LOQ; the LOQ represents the level at which a compound concentration was determined with sufficient precision. We defined the LOQ as three times the value of a blank sample, which was the blank matrix used in sample preparation. Detection rate was defined as percentage of samples with a detectable and quantifiable value. Because statistical handling of measurements below the LOD or below the LOQ may influence results, we tried different selection schemes to test whether conclusions were sensitive to the actual values of the unquantifiable measurements. We performed three analyses to compare the exposures of cryptorchid and normal boys. The first included all data using the LOD for samples with nondetectable values and the detected value for unquantified samples. The second included all data except for samples with values below the LOQ. The third analysis excluded all nondetectable samples. Exposure levels reported in Table 2 are based on the third analysis excluding samples with values below the LOD or below the LOQ. Exposure patterns between cases and controls using the other two statistical approaches did not substantially differ from those in Table 2 (data not shown).

To investigate a combined effect of persistent pesticides, we used eight pesticides for which all individuals had measurable and quantifiable levels [*p,p*′-DDE, *p,p*′-DDT, β-hexachlorocyclohexane (HCH), hexa-chlorobenzene (HCB), α -endosulfan, *cis*-heptachloroepoxide (*cis*-HE), oxychlordane, and dieldrin]. A statistical test of the null hypothesis that there were no differences in the median exposure levels of cases and controls was carried out as a Monte Carlo permutation test. In the permutations, cases and controls were randomly assigned exposure profiles from the observed profiles, and median levels between cases and controls were compared within countries. We then compared the observed test statistic (the number of median exposures, which were higher among cases than controls) with the permutation distribution. The permutation scheme of the test takes into account the within-individual association structure between different persistent organochlorine pesticides—that some individuals tend to have high exposure to many pesticides, whereas others have low exposures.

## Results

Study population characteristics are given in Table 1. We found no significant differences between the mothers giving birth to a cryptorchid boy versus a healthy boy. Gestational age of cryptorchid boys was slightly lower than in normal boys (Denmark: *p* = 0.029; Finland: *p* = 0.688). No systematic difference between cases and controls with regard to year of birth was observed (*p* = 0.543). In both countries, the selected controls did not differ from the healthy mothers and boys in the entire cohorts with respect to maternal age, parity, smoking, gestational age, and birth weight (data not shown).

Table 2 shows the results of pesticide measurements in breast milk samples of mothers with cryptorchid and normal boys. The lipid content (percent weight per weight) did not differ significantly (*p* = 0.707) between cases [3.7 (range, 1.1–7.9)] and controls [3.8 (range, 0.4–10.1)]. Concentrations of persistent pesticides (nanograms per gram lipid) in breast milk showed large interindividual variations and skewed distributions as well as differences in absolute levels.

Eight compounds—*p,p*′-DDE, β-HCH, HCB, α -endosulfan, oxychlordane, *p,p*′-DDT, dieldrin, and *cis*-HE (listed with decreasing concentrations)—were quantifiable in all samples. The concentrations of these eight compounds were higher than those of any of the remaining 19 compounds, which were all measured in very low concentrations. The sum of all DDT metabolites was slightly higher for cases than controls, but the difference did not reach statistical significance. The ratio between *p,p*′-DDE and *p,p*′-DDT was higher among controls than cases, but not significantly. Seventeen of 21 organochlorine pesticides [*p,p*′-DDT; *p,p*′-DDE; 1,1-dichloro-2,2-bis(4-chlorophenyl)ethane (*p,p*′-DDD); 1,1,1-trichloro-2-(2-chlorophenyl)-2-(4-chloropheny l)ethane (*o,p*′-DDT); HCH (α , β, γ ); HCB; pentachloroanisole (PCA); α -endosulfan; *cis*-HE; chlordane (*cis*-, *trans*-); oxychlordane; methoxychlor; octachlorostyrene (OCS); and dieldrin] were measured in slightly higher median concentrations in milk from mothers giving birth to cryptorchid boys than in mothers giving birth to healthy boys with only *trans*-chlordane reaching statistical significance (*p* = 0.012) (Table 2).

Table 3 presents the overall exposure pattern of cryptorchid and healthy boys depending on inclusion of samples with values below the LOD and/or LOQ. The overall exposure pattern in the three analyses was comparable, showing that most compounds were measured in higher concentrations in milk from case mothers than in milk from control mothers.

A combined analysis (a Monte Carlo permutation test) of the eight most prevalent organochlorine pesticides revealed that the difference between milk from case mothers and control mothers was not likely due to chance (*p* = 0.032).

OCS, pentachlorobenzene (PeCB), *p,p*′-DDD, *o,p*′-DDT, and mirex were detected in almost all samples (detection rates 90.8–99.2%), but at low concentrations. Aldrin, β-endosulfan, *trans*-heptachloroepoxide, and ɛ -HCH were not detected in any sample. Heptachlor and δ-HCH were measured at very low concentrations in three and five samples, respectively. None of these six pesticides are included in Table 2. For the remaining eight compounds [methoxychlor, *cis*-chlordane, PCA, γ -HCH, 1,1-dichloro-2-(2-chlorophenyl)-2,2(4-chlorophenyl)ethane (*o,p*′-DDD), *trans-*chlordane, α -HCH, 1,1-dichloro-2-(2-chlorophenyl)-2-(4-chlorophenyl)ethylene (*o,p*′-DDE)], the total detection rate varied between 26.9 and 71.5%. Detection rates did not differ significantly between cases and controls for single pesticides (data not shown).

For oxychlordane and *cis*-HE, the enantiomeric concentrations were available. The absolute concentrations of the enantiomeric isomers were higher, but not significantly, for cryptorchid boys than for controls (data not shown). The ER for oxychlordane (cases/controls) was 1.36/1.28. For *cis*-HE, the corresponding figures were 2.48/2.19 (*p* = 0.103 and *p* = 0.467, respectively).

## Discussion

Most persistent organochlorine pesticides were found in higher concentrations in boys with cryptorchidism than in controls, although no individual compound was significantly correlated with cryptorchidism. For eight chemicals (*p,p*′-DDE, *p,p*′-DDT, β-HCH, HCB, α -endosulfan, *cis*-HE, oxychlordane, and dieldrin), which were measurable in all samples, the differences between cases and controls were unlikely to be due to chance. Although we cannot exclude the possibility that individual chemicals alone may cause cryptorchidism, our study suggests that exposure to more than one chemical at low concentrations represents a risk factor for congenital cryptorchidism. There may also be simultaneous coexposure to other environmental chemicals that contribute to the effect on testicular descent; the pesticide measurements may represent a proxy marker (sentinel) for other exposures, such as brominated flame retardants.

We were able to quantify most organochlorine pesticides at low levels in breast-milk samples. This indicates that these chemicals are still relevant despite the fact that most have been banned or restricted in the study areas for many years. High DDE/DDT levels support the assumption the current exposure level primarily originates from previous contamination, environmental persistence, and long-range atmospheric dissipation, and less from imported food products and increased traveling activity to areas with ongoing use of pesticides such as DDT for malaria control.

Levels of the investigated pesticides have generally declined in the study area, whereas the birth prevalence of cryptorchidism appears to have increased (Boisen et al. 2004). This may explain why no single compound was strongly correlated with cryptorchidism. However, low concentrations of a mixture of chemicals over time may still be harmful to the fetus. In addition, the overall exposure to other chemicals with endocrine-disrupting activity may have increased in the same period. Thus, the persistent pesticides investigated here may reflect current overall exposure: Women with the highest levels of persistent pesticides may also be the ones with the highest concentrations of other endocrine-disrupting chemicals. This hypothesis is supported by a previous study that found the concentrations of HCB, *p,p*′-DDE, *p,p*′-DDT, and β-HCH in individual samples increased with increasing poly-chlorinated biphenyl concentrations (Andersen and Orbæk 1984).

To our knowledge, no previous studies have compared levels of persistent organochlo-rine pesticides in breast milk with the birth prevalence of cryptorchidism. Breast milk was chosen as a surrogate biomarker of previous maternal exposure to persistent pesticides because these compounds accumulate in lipid-rich tissue and thereby in breast milk (Beek 2000; Jensen and Slorach 1991). There is a dynamic equilibrium between levels of persistent compounds in maternal adipose tissue and breast milk (Cerrillo et al. 2005; Kanja et al. 1992; Rogan et al. 1986; Skaare et al. 1988; Waliszewski et al. 2001); therefore, daily intake of pesticides during lactation has little influence on the levels measured in milk. Both human and animal studies have demonstrated that pesticides during pregnancy can be transferred to the fetus by crossing the placenta (Foster et al. 2000; Lange et al. 2002; Waliszewski et al. 2000). Levels measured in breast milk were positively correlated to levels measured in umbilical cord samples (Cerrillo et al. 2005; Kanja et al. 1992; Skaare et al. 1988; Waliszewski et al. 2001). Concentrations of compounds in breast milk are a suitable proxy for fetal exposure during pregnancy. As persistent pesticides are accumulated in the lipid fraction of the breast milk, any variations in the lipid content may affect the levels measured. In our study, the women were carefully instructed to collect only hind milk, and they collected many small aliquots that were pooled over time. Thus, the breast milk samples in this study represent an average content over a long period. Because we found no differences in mean lipid content between cases and controls, the potential bias induced by lipid variation due to collection is negligible. In both countries, the selected controls did not differ from the healthy mothers and boys in the entire cohorts with respect to maternal age, parity, smoking, gestational age, and birth weight, and therefore we believe that the samples are representative.

Because of matching for the most common confounders, such as parity, in the Finnish population, there were no significant differences between cases and controls. In the Danish group, only gestational age differed slightly (*p* = 0.029). Primiparae, slim women, and smokers tend to have higher pesticide levels (Jensen and Slorach 1991). As more mothers of healthy boys were primiparae and nonsmokers and had lower mean body mass index (BMI) than mothers of cryptorchid boys, our study may underestimate the effect of organochlorine pesticide exposure on cryptorchidism. No definitive relation between maternal age and the level of organochlorine pesticides in breast milk has been reported; some studies have found that older mothers have higher levels, whereas others have not (Jensen and Slorach 1991). In our study, case mothers were slightly older than control mothers, but fewer of them were primiparae. Older mothers had higher concentrations [significant difference in three compounds (*p,p*′-DDE, oxychlordane, and α -endosulfan)]. However, older primiparae mothers [*n* = 7:3 cases (4.8%); 4 controls (5.9%)] especially contributed to this difference. Because they were equally distributed in the two groups, this cannot explain the difference between cases and controls. Milk from mothers giving birth to premature babies may be different in composition. One Danish study described higher levels of HCB in milk from mothers giving birth to premature infants (Jensen and Slorach 1991). In our study, levels were higher in milk from mothers giving birth to premature infants. The differences were not significant, and the number of premature infants was low (five cases and two controls) and cannot therefore explain the difference we found.

The sensitivity of the analytic method allowed the detection of traces of organochlorine pesticides below the LOQ. This phenomenon was, as expected, most frequent for pesticides detected in generally low concentrations. Because the statistical handling of these measurements can profoundly change the results, we evaluated our data carefully with different approaches and found that the overall findings remained unchanged.

Few other studies have investigated the possible relationship between persistent pesticide levels in biologic samples and the prevalence of cryptorchidism. Hosie et al. (2000) compared levels of pesticides [DDT and metabolites, toxaphene, HCH (α , β, γ ), HCB, PCA, PeCB, and several chlorinated cyclodienes such as heptachlor] in fat biopsies from 18 cryptorchid boys and 30 controls. Their findings were comparable with ours: Pesticide concentrations were higher among cases than controls, but reaching significance for only a few (HCB and heptachloroepoxide). However, the relatively limited total number of samples and especially the broad age range of boys (0.1–15 years) limits the interpretation of the data. For some participants, biomonitoring was conducted a long time after the relevant prenatal exposure window for testicular maldescent.

Two studies have been published comparing levels of DDE and DDT in maternal blood and cryptorchidism and hypospadias in the offspring based on two large birth cohorts conducted in the United States in the 1960s (Bhatia et al. 2005; Longnecker et al. 2002). Although both studies were based on biologic samples collected in a period during which DDT was still being used, neither study found firm associations. Bhatia et al. (2005) did not find any association. Longnecker et al. (2002) found adjusted odd ratios to be elevated, although not significantly, and concluded that the results were consistent with a modest to moderate association between DDE/DDT and cryptorchidism. Both studies were performed as nested case–control studies within large prospective birth cohort studies; the women were recruited during pregnancy and the diagnosis of cryptorchidism was well ascertained. Although these studies applied a different biologic matrix (blood) than ours (breast milk), the concentrations in ours were lower. This would also be expected, as the samples in both studies were collected in the period from 1959 to 1966, when DDT was still permitted.

Other studies without access to biologic samples have also reported associations between cryptorchidism and parental pesticide exposure (Garcia-Rodriguez et al. 1996; Garry et al. 1996; Kristensen et al. 1997; Pierik et al. 2004; Restrepo et al. 1990; Weidner et al. 1998). Most of these studies were register-based and retrospective. One was performed as a nested case–control study in a large birth cohort study comparing parental exposure (registered by interview of the parents after birth) among boys with cryptorchidism and hypospadias with the exposure of parents of normal boys (Pierik et al. 2004). Garcia-Rodriguez et al. (1996) conducted an ecologic study in which orchidopexy rates in different municipalities in Granada, Spain, were compared. Pesticide use was categorized into four groups; areas with high pesticide exposure had higher orchidopexy rates. Generally, the exposure assessment was indirect and based on job titles, pesticide purchase, or self-reported exposure with the possibility of recall bias in case–control studies. Distinction between maternal and paternal exposure is made only in some studies: Pierik et al. (2004) found that cryptorchidism was significantly associated with paternal pesticide exposure but not maternal. Weidner et al. (1998) described a significantly increased risk of cryptorchidism in sons of female gardeners. Finally, Restrepo et al. (1990) found increased risk with maternal pesticide exposure during pregnancy (relative risk = 4.6); however, this did not reach significance. Because most of these studies have been conducted recently, the pesticide exposure may reflect newer, nonpersistent pesticides. In addition, register data of cryptorchidism are less reliable because of variation in ascertainment and reporting (Toppari et al. 2001).

We found a large interindividual difference in concentrations of pesticides in breast milk. This was most likely related to individual differences in exposure and metabolism. Although no significant difference in factors such as lipid content in breast milk, maternal age, maternal BMI, parity, and smoking habits were found between the mothers of cryptorchid and healthy boys, these factors may have contributed to some of the interindividual differences (Jensen and Slorach 1991; Sim and McNeil 1992).

Compared with previous reports on Danish and Finnish breast milk, the average levels of persistent pesticides found in our study were low (Bro-Rasmussen et al. 1968; Jensen and Slorach 1991; Mussalo-Rauhamaa et al. 1988; Sundhedsstyrelsen 1999; Vuori et al. 1977; Wickstrom et al. 1983). This finding agrees with other reports on declining concentration of some persistent chemicals (Jensen and Slorach 1991; Noren and Meironyte 2000; Schade and Heinzow 1998; Solomon and Weiss 2002). The ERs indicated to what extent the chiral persistent pollutants were metabolized in the mother’s body after the exposure (Shen et al. 2006), for example, because (–)-*cis*-HE is more readily degraded than (+)-*cis*-HE in the human body; and if the two samples have the same levels of *cis*-HE with different ERs, the higher ER sample could have been exposed more heavily in the past. It is an interesting result that the case samples have higher levels of *cis*-HE with higher ERs (medians). This means, considering the already metabolized part, that case mothers should have been exposed to *cis*-HE more heavily than control mothers in their exposure histories.

The most recent data (1993–1994) from the Danish National Board of Health (DNBH) of breast milk samples from 86 primiparae 25–29 years of age showed median concentrations of 178, 38, 33, and 8 ng/g lipid for *p,p*′-DDE, β-HCH, HCB, and dieldrin, respectively (Sundhedsstyrelsen 1999). In our study, the corresponding figures were 135, 16, 12, and 5 ng/g lipid for primiparae of the same age. DNBH estimated that the average daily intake of *p,p*′-DDE by the child was 1.1 μg/kg/day; in our study, the corresponding value was 0.7 μg/kg/day. This value is still above the acceptable daily intake/tolerable daily intake (ADI/TDI) suggested in the same report (0.5 μg/kg/day) (Sundhedsstyrelsen 1999). For μ-HCH, HCB, and dieldrin, estimated average daily intake in our study (0.09, 0.07, 0.03 μg/kg/day) was below the recommended ADI/TDI (0.6, 0.17, and 0.05 μg/kg/day).

In contrast to a Finnish study from 1988 in which mirex was not detected in any of the milk samples (Mussalo-Rauhamaa et al. 1988), we were able to measure mirex in all samples, although in low concentrations. This difference could be due to our more sensitive analytic methods. To our knowledge, data on endosulfan, PCA, and PeCB in Danish and Finnish milk samples have not been published before. The levels were low compared with studies from other industrialized countries (Cerrillo et al. 2005; Mes et al. 1993; Newsome and Ryan 1999; Noren and Meironyte 2000).

In this study we focused on pesticides with suspected endocrine-disrupting activity or pesticides that have been used worldwide (European Commission Directorate General Environment 2000; Toppari et al. 1996). The relative potency of each pesticide is often much lower than that of natural hormones. However, several of the included compounds act as both estrogens and antiandrogens (Andersen et al. 2002; Kelce et al. 1995), which might increase their possible adverse effect on testicular descent. Furthermore, different compounds may interact, thereby enhancing their effects (Andersen et al. 2002; Kortenkamp and Altenburger 1998; LeBlanc et al. 1997; Silva et al. 2002). Existing data on possible mixture effects of the specific organochlorine pesticides *in vitro* are limited. A combination of 10 compounds including endosulfan*,* dieldrin, methoxychlor, and some DDT metabolites demonstrated a cumulative effect (Soto et al. 1994, 1995). Similarly, a number of studies have found indications of additivity for some of the pesticides included in our study (Arnold et al. 1997; Merritt et al. 1999; Payne et al. 2001; Shekhar et al. 1997; Sumpter and Jobling 1995; Vonier et al. 1996). Others have not been able to demonstrate additivity between dieldrin and endosulfan (Ashby et al. 1997; Wade et al. 1997) or methoxychlor (Ashby et al. 1997). Investigating the possible effects of mixtures is complicated because mechanisms of actions for individual compounds are often poorly known, and some chemicals may act through different routes depending on dosage.

In conclusion, our study suggests an association between congenital cryptorchidism and some persistent organochlorine pesticides present in mothers’ breast milk. Although our study cannot provide proof for a causal relationship, our data are in line with results from animal studies. Thus, prenatal exposure to persistent organochlorine pesticides may adversely affect testicular descent in boys.

## Figures and Tables

**Table 1:** Population characteristics among Danish and Finnish mothers giving birth to cryptorchid boys and healthy boys in a joint Danish and Finnish case–control study.

	Denmark			Finland		
Characteristic	Cases (*n* = 29)	Controls (*n* = 36)	*p*-Value	Cases (*n* = 33)	Controls (*n* = 32)	*p*-Value
Maternal age (years)	31.0 ± 3.9	30.8 ± 4.4	0.908	29.8 ± 4.9	28.8 ± 4.7	0.408
Maternal height (cm)	170.3 ± 5.1	170.1 ± 6.0	0.896	165.9 ± 5.4	166.5 ± 4.5	0.634
Prepregnancy weight (kg)	67.9 ± 8.5	67.2 ± 8.9	0.739	65.0 ± 10.7	61.1 ± 6.4	0.083
Prepregnancy BMI	23.4 ± 3.3	23.2 ± 3.0	0.860	23.7 ± 4.0	22.1 ± 2.3	0.056
Parity [*n* (%)]						
1	20 (69.0)	28 (77.8)		17 (51.5)	19 (59.4)	
2	5 (17.2)	4 (11.1)	0.705	9 (27.3)	9 (28.1)	0.633
≥ 3	4 (13.8)	4 (11.1)		7 (21.2)	4 (12.5)	
Smoking [*n* (%)]	8 (28.6)	11 (30.6)	0.863	5 (15.6)	5 (15.6)	1.000
Gestational age (days)	276 ± 15	283 ± 8	0.029	278 ± 11	280 ± 9	0.688
Birth weight (kg)	3.6 ± 0.7	3.8 ± 0.5	0.135	3.6 ± 0.5	3.6 ± 0.4	0.506
Birth length (cm)	52.3 ± 3.8	53.1 ± 2.1	0.246	51.2 ± 1.9	51.1 ± 1.9	0.956

Values are given as mean ± SD or number (%). The *p*-value describes differences between cases and controls.

**Table 2:** Pesticide concentrations in human breast milk samples from mothers of 62 boys with cryptorchidism and 68 healthy boys.^a^

				Lipid-based concentrations (ng/g lipid)				
	Detection rate (%)	Samples < LOD (*n*)	Samples < LOQ (*n*)	Cases (*n* = 62)	Controls (*n* = 68)	*p*-Value unadjusted	*p*-Value adjusted^b^	*p*-Value adjusted^c^
Lipid (% w/w)	100	0	0	3.68 (1.13–7.87)	3.76 (0.36–10.14)	0.52	0.71	0.79
*p,p*′-DDT	100	0	0	4.63 (1.85–14.26)	3.98 (1.46–37.88)	0.53	0.34	0.47
*p,p*′-DDE	100	0	0	97.32 (21.77–427.55)	83.76 (18.95–377.49)	0.70	0.40	0.26
*p,p*′-DDD	97.7	0	3	0.36 (0.11–1.88)	0.34 (0.10–2.20)	0.68	0.57	0.48
*o,p*′-DDT	98.5	0	2	0.35 (0.10–1.80)	0.34 (0.07–1.83)	0.50	0.68	0.97
*o,p*′-DDE	71.5	0	37	0.08 (0.03–0.28)	0.08 (0.03–0.20)	0.79	0.75	0.68
*o,p*′-DDD	53.1	3	58	0.03 (0.02–0.07)	0.03 (0.00–0.17)	0.60	0.65	0.36
*p,p*′-DDE/*p,p*′-DDT				17.88 (8.10–54.49)	19.31 (4.36–106.66)	0.80	0.94	0.59
Sum DDT				140.41 (43.04–442.70)	116.60 (30.72–245.17)	0.71	0.40	0.27
α-HCH	69.2	0	40	0.20 (0.04–3.45)	0.19 (0.04–2.36)	0.66	0.93	0.77
β-HCH	100	0	0	13.64 (2.74–66.23)	12.29 (4.47–59.30)	0.40	0.19	0.16
γ-HCH	45.4	0	71	[Table-fn tfn2-ehp0114-001133]— (0.36–4.05)	[Table-fn tfn2-ehp0114-001133]— (0.29–2.37)	0.13	0.06	0.77
HCB	100	0	0	10.59 (3.18–23.00)	8.83 (2.94–24.56)	0.38	0.15	0.12
PCA	43.1	1	73	[Table-fn tfn2-ehp0114-001133]— (0.03–0.79)	[Table-fn tfn2-ehp0114-001133]— (0.02–0.68)	0.34	1.00	0.78
PeCB	91.5	0	11	0.27 (0.11–1.41)	0.28 (0.08–0.61)	0.08	0.05	0.04
α-Endosulfan	100	0	0	6.95 (1.83–17.84)	6.66 (1.19–22.66)	0.13	0.10	0.06
*cis*-HE	100	0	0	2.48 (0.66–10.82)	2.19 (0.63–17.02)	0.26	0.16	0.36
*cis*-Chlordane	30.8	66	24	[Table-fn tfn2-ehp0114-001133]— (0.02–0.07)	[Table-fn tfn2-ehp0114-001133]— (0.01–0.07)	0.06	0.05	0.01
*trans*-Chlordane	61.5	22	28	0.06 (0.02–0.35)	0.04 (0.01–0.13)	0.01	0.01	0.01
Oxychlordane	100	0	0	4.52 (0.91–8.93)	4.09 (1.14–12.01)	0.71	0.51	0.21
Methoxychlor	26.9	1	94	[Table-fn tfn2-ehp0114-001133]— (0.06–1.12)	[Table-fn tfn2-ehp0114-001133]— (0.05–0.41)	0.37	0.30	0.17
OCS	90.8	0	12	0.21 (0.04–0.49)	0.18 (0.05–0.70)	0.15	0.14	0.78
Dieldrin	100	0	0	4.06 (0.77–35.50)	3.11 (1.07–14.05)	0.30	0.08	0.28
Mirex	99.2	0	1	0.21 (0.05–1.54)	0.23 (0.02–0.66)	0.92	0.97	0.67

—, Median cannot be determined.

a Pesticide concentrations (nanograms per gram lipid) are given as medians (for compounds with detection rate > 50%) and range (minimum to maximum), detection rates as percentages (%). Differences between cases and controls were tested by logistic regression.

b *p*-Value adjusted for country in a regression model.

c *p*-Value adjusted for country, maternal age, gestational age, parity, birth weight, and maternal prepregnancy BMI in a logistic regression model.

**Table 3:** Exposure pattern of cryptorchid versus healthy boys described by three statistical approaches.

		No. of compounds for which the median pesticide concentration (ng/g lipid) is:		
Type of samples included in the analysis	No. of pesticides included	Cases > controls	Cases = controls	Cases < controls
I^a^	23	14	5	4
II^b^	23	15	5	3
III^c^	21^d^	17	2	2

a All samples are included, samples below the LOD as the LOD values and samples below the LOQ with the measured value.

b Samples below the LOD included as the LOD values, samples < LOQ excluded.

c Only measurements above the LOD and above the LOQ are included.

d Heptachlor and δ-HCH not included.
